# Comparison between external fixation and pelvic binder in patients with pelvic fracture and haemodynamic instability who underwent various haemostatic procedures

**DOI:** 10.1038/s41598-022-07694-3

**Published:** 2022-03-07

**Authors:** Ji Young Jang, Keum Soek Bae, Byung Hee Kang, Gil Jae Lee

**Affiliations:** 1 Department of Surgery, National Health Insurance Service Ilsan Hospital, Goyang, Republic of Korea; 2 Department of Surgery, Yonsei University Wonju College of Medicine, Wonju, Republic of Korea; 3 Department of Surgery, Ajou University School of Medicine, 164 World cup-ro, Yeongtong-gu, Suwon, Gyeonggi-do 16499 Republic of Korea; 4 Department of Traumatology, Gachon University College of Medicine, 38-13, Dokjeom-ro 3beon-gil, Namdong-gu, Incheon, 21565 Republic of Korea

**Keywords:** Trauma, Outcomes research, Medical research

## Abstract

Haemostatic procedures such as preperitoneal pelvic packing (PPP), pelvic angiography (PA), and internal iliac artery ligation are used for haemorrhage control in pelvic fracture patients with haemodynamic instability. Pelvic external fixation (PEF) and pelvic binder (PB) are usually applied with haemostatic procedures to reduce the pelvic volume. This study aimed to compare the clinical outcomes between patients who underwent PEF and PB. Among 173 patients with pelvic fracture admitted to the emergency room of three regional trauma centres between January 2015 and December 2018, the electronic charts of haemodynamically unstable patients were retrospectively analysed. Among the 84 patients included in the analysis, 20 underwent PEF with or without PB, and 64 underwent only PB. There were significant differences in tile classification and laparotomy between the PEF and PB groups (*p* = 0.023 and *p* = 0.032). PPP tended to be more frequently preformed in the PEF group (*p* = 0.054), whereas PA tended to be more commonly performed in the PB group than in the PEF group (*p* = 0.054). After propensity score matching to adjust for differences in patient characteristics and adjunct haemostatic procedure, there was no significant difference in 7-day, 30-day, and overall mortality rates between the PEF and PB groups (10.5% vs 21.1%, *p* = 0.660, 21.1% vs 26.3%, *p* = 1.000, and 26.3% vs 26.3%, *p* = 1.000). Cox proportional hazard regression analysis and multivariate analysis for correction of covariates (age, lactate, and abdominal injury) showed that PEF was not an independent factor for 30-day mortality compared with PB (adjusted hazard ratio, 0.526; 95% confidence interval, 0.092–3.002; *p* = 0.469). Among the volume reduction procedures performed with other haemostatic procedures in patients with pelvic fracture and haemodynamic instability, PEF did not significantly reduce the 30-day mortality rate compared to PB.

## Introduction

Despite advances in haemostatic procedures for patients with haemodynamic instability and pelvic fractures, the mortality rate among them is high^1–4^. Haemorrhage is the most common cause of death in such patients, and internal iliac artery ligation, pelvic angiography (PA), preperitoneal pelvic packing (PPP), and resuscitative endovascular balloon occlusion of the aorta (REBOA) are used in various combinations for achieving haemostasis^3,5–9^. Early pelvic stabilisation reduces the pelvic cavity due to fracture and induces retroperitoneal tamponade to reduce bleeding and prevent further pelvic damage. Recently, pelvic external fixation (PEF) and pelvic binder (PB) have been mainly used as damage control orthopaedic techniques in patients with pelvic fracture and haemodynamic instability in the acute phase^2,10,11^. In addition, a pelvic orthotic device has been developed and marketed that can be conveniently used in various emergency centres for patients with unstable pelvic fractures (T-POD, Morrisville, NC, US)^11^. Although various studies on the application of PPP and PA in such patients have been published recently, limited studies have compared PEF and PB^8,12–14^. Therefore, this study aimed to compare clinical outcomes between patients who underwent PEF and PB for pelvic volume reduction.

## Results

### Patient characteristics

The average patient age was 54.1 ± 16.3 years, and the proportion of males was 69.0%. The most common injury mechanism was fall from a height (34.5%), followed by pedestrian traffic accidents (33.3%) and crushes (11.9%). In total, 53.6% and 45.2% patients were classified into types B and type C according to tiles classification. The average systolic blood pressure was 95.7 ± 28.3 mmHg, and the average initial lactate level was 5.22 ± 3.14 mmol/L. REBOA and PPP were performed in 9 (10.7%) and 43 (51.2%) patients, respectively. Laparotomy was performed in 12 patients (14.3%), and arterial embolization was performed in 28 (68.3%) of 41 patients who underwent PA. Furthermore, 14 (16.7%) patients underwent PEF after PB, six (7.1%) underwent PEF without PB, and 64 (76.2%) underwent only PB. Among the associated injuries (AIS > 3), chest injury was the most common, followed by head and neck and abdominal injuries. The mean injury severity score (ISS) was 38.9 ± 12.0. The 7-day, 30-day, and overall mortality rates were 9.5%, 20.2%, and 22.6%, respectively (Table 1).

**Table 1:** Patient characteristics.

Variables	*N* = 84
Age	54.1 ± 16.3
Sex (male)	58 (69.0)
**Injury mechanism**	
Fall	29 (34.5)
Motor vehicle crash	5 (6.0)
Motorcycle	7 (8.3)
Pedestrian traffic accident	28 (33.3)
Crushing	10 (11.9)
Other	5 (6.0)
**Title classification**	
A	1 (1.2)
B	45 (53.6)
C	38 (45.2)
Open fracture	4 (4.8)
Initial systolic blood pressure	95.7 ± 28.3
Initial haemoglobin	10.2 ± 2.7
Initial lactate	5.22 ± 3.14
Initial lactate > 4 mmol/L	44 (52.4)
REBOA	9 (10.7)
PPP	43 (51.2)
Laparotomy	12 (14.3)
Pelvic angiography/embolization	41 (48.8)/28 (68.3)
Pelvic external fixation	20 (23.8)
With pelvic binder	14
Without pelvic binder	6
Pelvic binder only	64 (76.2)
OR and IF	55 (65.5)
**Combined injury**	
Head or neck injury (AIS > 3)	13 (15.5)
Chest injury (AIS > 3)	14 (16.7)
Abdomen injury (AIS > 3)	8 (9.5)
ISS	38.9 ± 12.0
Requirement of RBC for 4 h	7 (0–41)
Requirement of RBC for 24 h	15.5 (0–114)
ICU stay	11.5 (0–256)
Hospital stay	46 (2–315)
7 day mortality	8 (9.5)
30 day mortality	17 (20.2)
Overall mortality	19 (22.6)

*REBOA* resuscitative endovascular balloon occlusion of the aorta, *PPP* preperitoneal pelvic packing, *OR and IF* open reduction and internal fixation, *AIS* Abbreviated Injury Scale, *ISS* Injury severity score, *RBC* red blood cell, *ICU* intensive care unit.

### PEF group versus PB group before PSM

There were significant differences in the injury mechanism, tile classification, and laparotomy between the PEF and PB groups (*p* = 0.022, *p* = 0.023, and *p* = 0.032, respectively). PPP tended to be more frequently performed in the PEF group than in the PB group (*p* = 0.054), whereas PA tended to be more commonly performed in the PB group than in the PEF group (*p* = 0.054; Table 2).

**Table 2:** Comparison between the pelvic external fixation group and the pelvic binder only group.

Variables	PEF group *N* = 20	Pelvic binder only group *N* = 64	*p* value
Age	54.8 ± 17.7	53.9 ± 15.9	0.847
Sex (male)	16 (80.0)	42 (65.6)	0.225
Injury mechanism			0.022*
Fall	2 (10.0)	27 (42.2)	
Driver	3 (15.0)	2 (3.1)	
Motorcycle	3 (15.0)	4 (6.3)	
Pedestrian	7 (35.0)	21 (32.8)	
Crushing	4 (20.0)	6 (9.4)	
Other	1 (5.0)	4 (6.3)	
Tile classification			0.023*
A	0	1 (1.6)	
B	6 (30.0)	39 (60.9)	
C	14 (70.0)	24 (37.5)	
Initial systolic blood pressure	98.7 ± 31.5	94.8 ± 27.5	0.591
Initial haemoglobin	9.9 ± 2.5	10.3 ± 2.8	0.542
Initial lactate	5.31 ± 3.26	5.20 ± 3.13	0.893
Combined Injury			
Head or neck Injury (AIS > 3)	2 (10.0)	11 (17.2)	0.724*
Chest injury (AIS > 3)	1 (5.0)	13 (20.3)	0.171*
Abdomen injury (AIS > 3)	1 (5.0)	7 (10.9)	0.673*
ISS > 25	17 (85.0)	54 (84.4)	1.000*
REBOA	3 (15.0)	6 (9.4)	0.439*
PPP	14 (70.0)	29 (45.3)	0.054
Pelvic angiography	6 (30.0)	35 (54.7)	0.054
Laparotomy	6 (30.0)	6 (9.4)	0.032
OR and IF	13 (65.0)	42 (65.6)	0.959
Requirement of RBC for 4 h	8.5 (2–32)	7 (0–41)	0.373
Requirement of RBC for 24 h	24.5 (3–114)	12.5 (0–94)	0.137
ICU stay	15 (0–160)	11 (0–256)	0.525
Hospital stay	70.5 (4–315)	41 (2–260)	0.135
7-day mortality	2 (10.0)	6 (9.4)	1.000*
30-day mortality	4 (20.0)	13 (20.3)	1.000*
Overall mortality	5 (25.0)	14 (21.9)	0.766*

*AIS* Abbreviated Injury Scale, *ISS* injury severity score, *REBOA* resuscitative endovascular balloon occlusion of the aorta, *PPP* preperitoneal pelvic packing, *OR and IF* open reduction and internal fixation, *RBC* red blood cell, *ICU* intensive care unit, *PEF* pelvic external fixation.

### PEF group versus PB group after PSM

One-to-one PSM was performed for four variables—tile classification, PPP, PA, and laparotomy. After PSM, these variables were similar between the PEF and PB groups. Nineteen patients were eligible for PSM. For a more objective evaluation of the characteristic balance, we calculated the standardised differences of the selected confounders. We observed a small effect size for all covariates, defined by a standardised difference value below 0.2 after matching (Table 3).

**Table 3:** Standardized difference.

Variables	PB group (*N* = 19)	PEF group (*N* = 19)	Standardized difference*
**Tile’s classification**			
B	5 (26.32)	6(31.58)	− 0.11625
C	14(73.68)	13(68.42)	
PPP	14(73.68)	13(68.42)	− 0.11625
Laparotomy	4(21.05)	5(26.32)	0.12403
Pelvic angiography	6(31.58)	5(26.32)	− 0.11625

*Standardized difference: difference in means or proportions divided by standard error; imbalance defined as absolute value greater than 0.20 (small effect size).

*PB* pelvic binder, *PEF* pelvic external fixation, *PPP* preperitoneal pelvic packing.

There was no significant difference in the 30-day mortality rate between the two groups (PEF group: 21.1% vs. PB group: 26.3%, *p* = 1.000). There was also no difference in the 7-day mortality rate and overall mortality rate between the two groups (7-day mortality rate: 10.5% vs. 21.1%, *p* = 0.660; overall mortality rate: 26.3% vs. 26.3%, *p* = 1.000). There were no significant differences in RBC requirement between the PEF and PB groups at 4 h and 24 h (*p* = 0.612 and *p* = 0.917, respectively; Table 4).

**Table 4:** Comparison between the pelvic external fixation group and pelvic binder group after propensity score matching.

Variables	PEF group *N* = 19	Pelvic binder group *N* = 19	p-value
Age	56.0 ± 17.3	47.7 ± 14.6	0.121**
Sex (male)	15 (78.9)	11 (57.9)	0.163
Injury mechanism			0.093*
Fall	2 (10.5)	8 (42.1)	
Driver	3 (15.8)	2 (10.5)	
Motorcycle	3 (15.8)	2 (10.5)	
Pedestrian	7 (36.8)	6 (31.6)	
Crushing	4 (21.1)	0	
Other	0	1 (5.3)	
Tile classification			0.721
B	6 (31.6)	5 (26.3)	
C	13 (68.4.0)	14 (73.7)	
Young and Burges classification			0.365*
APC type II	4 (21.1)	3 (15.8)	
APC type III	3 (15.8)	1 (5.3)	
LC type II	3 (15.8)	1 (5.3)	
LC type III	1 (5.3)	0	
VS type	7 (36.8)	12 (63.2)	
Other types	1 (5.3)	2 (10.6)	
Initial systolic blood pressure	98.1 ± 32.2	98.0 ± 33.3	0.996**
Initial haemoglobin	9.9 ± 2.6	11.4 ± 3.1	0.124**
Initial lactate	5.20 ± 3.32	7.43 ± 3.90	0.066**
Combined Injury			
Head or neck Injury (AIS > 3)	2 (10.5)	5 (26.3)	0.405*
Chest injury (AIS > 3)	1 (5.3)	7 (36.8)	0.042*
Abdomen injury (AIS > 3)	1 (5.3)	4 (21.1)	0.340*
ISS > 25	16 (84.2)	18 (94.7)	0.604*
REBOA	2 (10.5)	3 (15.8)	1.000*
PPP	13 (68.4)	14 (73.7)	0.721
Pelvic angiography	5 (26.3)	6 (31.6)	0.721
Laparotomy	5 (26.3)	4 (21.1)	1.000*
OR and IF	12 (63.2)	13 (68.4)	0.732
Requirement of RBC for 4 h	10 (2–32)	10 (0–41)	0.612**
Requirement of RBC for 24 h	23 (3–114)	24 (4–94)	0.917**
ICU stay	14 (0–160)	15 (0–92)	0.634**
Hospital stay	65 (4–315)	53 (2–260)	0.736**
7-day mortality	2 (10.5)	4 (21.1)	0.660*
30-day mortality	4 (21.1)	5 (26.3)	1.000*
Overall mortality	5 (26.3)	5 (26.3)	1.000

*APC* anterior–posterior compression, *LC* lateral compression, *VS* vertical shear, *AIS* Abbreviated Injury Scale, *ISS* injury severity score, *REBOA* resuscitative endovascular balloon occlusion of the aorta, *PPP* preperitoneal pelvic packing, *OR and IF* open reduction and internal fixation, *RBC* red blood cell, *ICU* intensive care unit, *PEF* pelvic external fixation.

*Result of Fisher’s exact test.

**Result of the Mann–Whitney U test.

Furthermore, Cox proportional hazard regression analysis and multivariate analysis for correction of covariates (age, lactate, and abdominal injury) showed that PEF was not an independent factor for 30-day mortality compared with PB (adjusted hazard ratio, 0.526; 95% confidence interval, 0.092–3.002; *p* = 0.469; Table 5).

**Table 5:** Cox proportional hazard ratio analysis for the 30-day mortality rate.

Variable	Crude hazard ratio	*P* value	Adjusted hazard ratio	*p* value
Age	1.043 (1.000–1.089)	0.051	1.111 (1.026–1.202)	0.009
Lactate	1.161 (0.983–1.371)	0.079	1.409 (1.085–1.832)	0.010
Abdomen injury (AIS > 3)	5.357 (1.322–21.701)	0.019	7.387 (1.444–37.801)	0.016
PEF	0.738 (0.198–2.748)	0.650	0.526 (0.092–3.002)	0.469

*AIS* Abbreviated Injury Scale, *PEF* pelvic external fixation.

## Discussion

Recent studies have reported that PEF plays an important role in haemostasis through stabilisation of the pelvic ring in pelvic fracture with shock to reduce additional damage and the reduction effect of the pelvic cavity^11–13^. However, a recent study that analysed 10-year data on external emergent stabilisation using the German pelvic trauma registry showed a decreasing trend in the use of PEF in patients with pelvic ring fracture. In contrast, the use of PB has increased rapidly, and it was used in almost 40% patients^10^. Moreover, in a recent multi-centre study conducted in a level I trauma centre in the United States, PB was performed in 50% patients with pelvic fracture and shock, and PEF was performed in only 4% patients^12^. These results show that PB has been increasingly used instead of PEF, and its use has been continuously increasing due to its simplicity and speed in application^11,15^. It is difficult to compare the effects of PEF and PB in the treatment of patients with haemorrhage due to pelvic fracture compared to haemorrhage due to other injuries because a combination of various modalities is possible. In 2007, in a comparative study between the PEF and PB groups, Croce et al. showed that the mortality rates were similar, but the requirement for packed RBC transfusion at 24 and 48 h was significantly lower in the PB group than in the PEF group. However, there was a difference in characteristics between the two groups. Since the recently used procedures such as PPP or REBOA were not analysed together, it is difficult to accept the results in the current scenario^11^. In our study, to minimise the effect of other haemostatic procedures and compare the differences between the effects of PB and PEF, the proportions of patients who had undergone PPP, PA, and laparotomy were corrected using PSM. The results showed that there were no significant differences in 7-day, 30-day, and overall mortality rates between the PEF and PB groups. Recent studies have recommended that PB be applied as soon as possible after injury for rapid volume reduction of the pelvic cavity^16,17^. This means that haemostatic modalities such as PPP, PA, and REBOA should be used as much as possible to stop haemorrhage, the cause of most deaths, and simultaneously, a procedure for volume reduction of the pelvic cavity applied as quickly as possible should be performed. There was no difference in clinical outcome between the two groups in the present study because six of 20 patients in the PEF group underwent PEF together with rapid PPP. In 14 patients, PB was immediately applied in the ER and then removed immediately before PEF. Hence, the haemostatic effect by volume reduction of the pelvic cavity in the acute phase was evidently similar between the two groups.

If PB is not removed quickly or over-tightened, complications such as skin necrosis and pressure ulceration may occur; therefore, it is recommended that PB be maintained for < 24–48 h^2,18^. In the trauma centres included in this study, PB was removed within 48 h when the patient was haemodynamically stabilised; however, the definitive fixation of the pelvis was determined considering the patient’s condition and was performed after an average of 6 days after injury. This suggests that pelvic volume reduction does not significantly affect the patient’s outcome after acute haemostasis, and the results of our study are consistent with the recent trend in which the use of PB rather than PEF is continuously increasing.

In addition, the Denver group reported that patients with pelvic fracture and haemodynamic instability undergoing PEF with PPP had a very good overall mortality rate^5,13^. However, it is difficult to explain this result only with the effect of PEF application; the protocolized multi-disciplinary approach for pelvic fracture with shock, application of the critical pathway, and active use of PPP apparently acted in combination. The pelvic trauma management algorithm of the World Society of Emergency Surgery was used to define severe lesions (WSES grade IV) regardless of mechanical instability in cases of haemodynamic instability. After application, haemostatic procedures such as PPP, mechanical fixation, REBOA, and PA should be performed complementarily^2^. In our study, before PSM, PPP with PEF was most commonly performed in the PEF group (70.0%), whereas PA was most commonly performed in the PB group (54.7%). To overcome this tendency of the combination of haemostatic procedures and to confirm the pure effect of PEF application, the ratio of haemostatic procedures (PPP, PA, and laparotomy) applied together between the two groups was corrected by PSM.

REBOA has recently been increasingly used in patients with haemodynamic instability instead of emergent resuscitative thoracotomy^19–22^. In Korea, REBOA was first used in regional trauma centres in 2016. It is being used as a bridge procedure before other haemostasis in patients with pelvic fracture accompanied by severe shock^22^. In our study, nearly all patients underwent both the PPP and PA procedures (88.9%). The 7-day and 30-day mortality rates in these patients were 22.2% and 44.4%, respectively, and PEF was performed in only three patients. These results are thought to be because REBOA was used in patients with a clinically critical condition, and PB, which can be easily applied, was preferred over PEF when it was necessary to move to the operating room. Although patients were not matched according to REBOA application by PSM, the application rate between the two groups was the same after PSM; therefore, it is judged that the effect of REBOA application did not affect the clinical outcome.

Our study has certain limitations. First, since this was a retrospective study, selection bias was observed between the two groups. Second, the statistical power was low because the number of patients who underwent PEF was very small. Third, in the PEF group, only six patients received PEF without PB, and the remaining patients received PEF after PB application. Therefore, strictly speaking, it is difficult to claim that our study compared the haemostatic effects of PEF and PB in the acute phase. However, it is difficult to conduct a randomised controlled trial to compare the effects of PB and PEF on haemostasis in the acute phase. In addition, since most of the patients included in our study were those who received PB in the ER, we focused on confirming the effect of PEF after the acute phase. For this reason, patients who died within 24 h in this study were excluded from the analysis. Nevertheless, this study is rare on the effectiveness of pelvic stabilisation procedures performed with various haemostatic procedures in patients with haemodynamic instability and pelvic fractures. The advantages of this study are that PSM was performed to correct for various confounding factors, and that patients from three institutions were included in the study. In the future, a larger prospective study is needed to confirm the results of our study.

## Methods

### Study setting

Wonju Severance Christian Hospital, Ajou University Hospital, and Gachon University Gil Medical Center participated in this study. All three hospitals are regional trauma centres designated and supported by the Ministry of Health and Welfare of Korea. These hospitals operate in accordance with the American Association for the Surgery of Trauma Level 1 trauma centre standards in terms of facilities, equipment, personnel, and operations.

**Figure 1:**
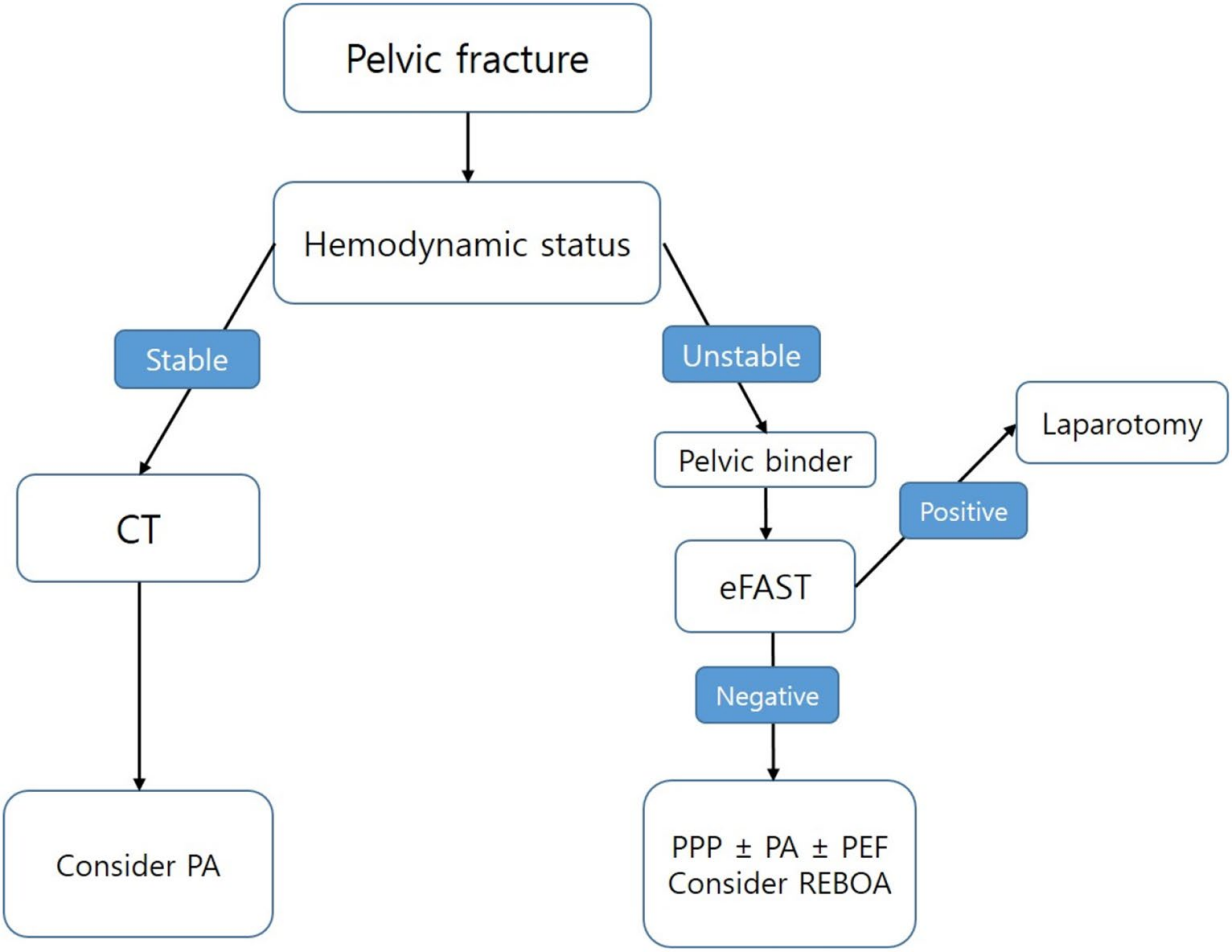
Pelvic fracture management algorithm. *CT* computed tomography, *eFAST* extanded focused assessment with sonography for trauma, *PPP* preperitoneal pelvic packing, *PA* pelvic angiography, *PEF* pelvic external fixation, *REBOA* resuscitative endovascular balloon occlusion of the aorta.

This study was approved by Gil Medical Center Institutional Review Board (IRB) (No. GCIRB2021-111), which waived the requirement for informed consent due to the retrospective nature of the study. This study was conducted according to the guidelines of the Declaration of Helsinki. Patients with haemodynamic instability underwent pelvic AP radiography and extended focused assessment with sonography for trauma as soon as they arrived at the trauma resuscitation room; if pelvic fracture was found to be the main cause of bleeding with instability of the pelvic ring, a trauma pelvic orthotic device (T-POD) was applied. Depending on the patient’s condition, the trauma surgeon decided to apply haemostatic procedures such as REBOA, PPP, and PA. Moreover, the application of PEF was decided after consultation with trauma and orthopaedic surgeons in charge of the trauma department. T-POD was used as PB in all hospitals included in our study. When other haemostatic procedures were performed, it was released immediately before the procedure and reapplied immediately after the completion of the procedures. All three hospitals have similar pelvic fracture management algorithms, summarised as follows (Fig. 1).

### Study population

**Figure 2:**
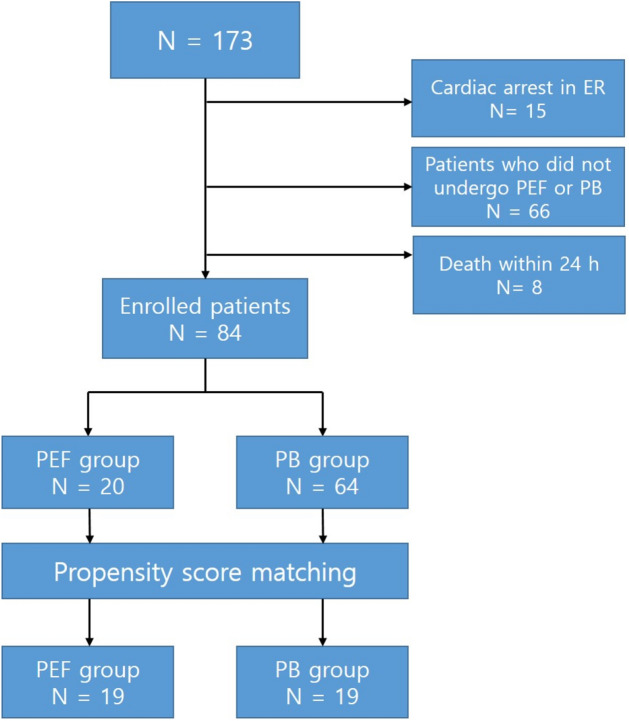
Patient flowchart.

Among the patients with pelvic fractures who visited the trauma resuscitation room from January 2015 to December 2018, patients with an Abbreviated Injury Scale (AIS) score of ≥ 4 points and a blood pressure of < 90 mmHg or blood lactate level of ≥ 2 mmol/L in the emergency room (ER) were included in the study. Patients who had more haemorrhages due to causes other than pelvic fractures and those without haemorrhagic shock were excluded. After screening, 173 patients were included in the study. Among them, 66 patients who did not undergo PEF or PB for pelvic cavity reduction, 15 patients who developed cardiac arrest in the ER, and 8 patients who died within 24 h of ER arrival were excluded. Consequently, 84 patients were included in the study. Regardless of whether PB was applied, the patients were divided into two groups, the PEF group, and the PB group, depending on whether or not PEF was applied. Twenty patients in the PEF group were compared with 64 patients in the PB group (Fig. 2).

### Outcome measures

The primary outcome was the 30-day mortality rate. Hospital mortality rate, 7-day mortality rate, requirements of packed red blood cells (RBCs) for 24 h, length of intensive care unit (ICU) stay, and duration of hospitalisation were evaluated as the secondary outcomes.

### Statistical analysis

Continuous variables, expressed as mean ± standard deviation or median (minimum–maximum), were analysed using Student’s t-test or the Mann–Whitney U test. Categorical variables were analysed using the chi-square test or Fisher’s exact test. Logistic regression analysis estimated the propensity score model, and the probability that each subject will be included in the control group by the given covariance corresponds to the propensity score. We performed nearest-neighbor matching (caliper distance: 0.25). This method matching method, the absolute values of the differences in the estimated propensity scores of all patients in the PEF group and PB group were paired from smallest to largest. The C-statistic of the logistic regression model for propensity score matching (PSM) was 0.790. The covariates included in the calculation were tile classification, PPP, laparotomy, and PA (*p* < 0.1). Cox proportional hazard regression analysis was performed as a multivariate analysis to compare the 30-day mortality rate corrected for covariates between the two groups. Statistical significance was set at *p* < 0.05. Statistical analyses were performed using IBM SPSS ver. 23.0 (IBM Inc., Armonk, NY, USA) and SAS 9.4 software (SAS Inc., Cary, NC, USA).
